# Research advances in kinase enzymes and inhibitors for cardiovascular disease treatment

**DOI:** 10.4155/fsoa-2017-0010

**Published:** 2017-08-08

**Authors:** Rand Shahin, Omar Shaheen, Faris El-Dahiyat, Maha Habash, Sana Saffour

**Affiliations:** 1 Department of Pharmaceutical Chemistry, Faculty of Pharmaceutical Sciences, Hashemite University, Az-Zarqa, Jordan; 2 Department of Pharmacology, Faculty of Medicine, University of Jordan, Amman, Jordan; 3 Department of Clinical Pharmacy & Pharmacy Practice, Faculty of Pharmaceutical Sciences, Hashemite University, Az-Zarqa, Jordan; 4 Department of Pharmaceutical Sciences & Pharmacognosy, Faculty of Pharmacy, Aqaba University of Techonology, Aqaba, Jordan; 5 Faculty of Pharmaceutical Sciences, Hashemite University, Az-Zarqa, Jordan

**Keywords:** CaMK-II, cardiovascular, GSK-3β, inhibitor, kinase inhibitor, MAPK, PKC, protein kinases, ROCK II

## Abstract

The targeting of protein kinases has great future potential for the design of new drugs against cardiovascular diseases (CVDs). Enormous efforts have been made toward achieving this aim. Unfortunately, kinase inhibitors designed to treat CVDs have suffered from numerous limitations such as poor selectivity, bad permeability and toxicity. So, where are we now in terms of discovering effective kinase targeting drugs to treat CVDs? Various drug design techniques have been approached for this purpose since the discovery of the inhibitory activity of Staurosporine against protein kinase C in 1986. This review aims to provide context for the status of several emerging classes of direct kinase modulators to treat CVDs and discuss challenges that are preventing scientists from finding new kinase drugs to treat heart disease.

## Lay abstract

Various protein kinase enzymes are distinctly involved in diverse signal pathways involved in regulating cardiac function in health and disease states. In 1986, the inhibitory activity of Staurosporine against protein kinase C was revealed, and since then, the interest in finding active kinase inhibitors for use in medicine has grown. Numerous kinase inhibitors have been synthesized in the last two decades, yet none has found its way to the market as a cardiovascular disease treatment; thus, more scientific efforts are needed in this regard.

## Graphical abstract

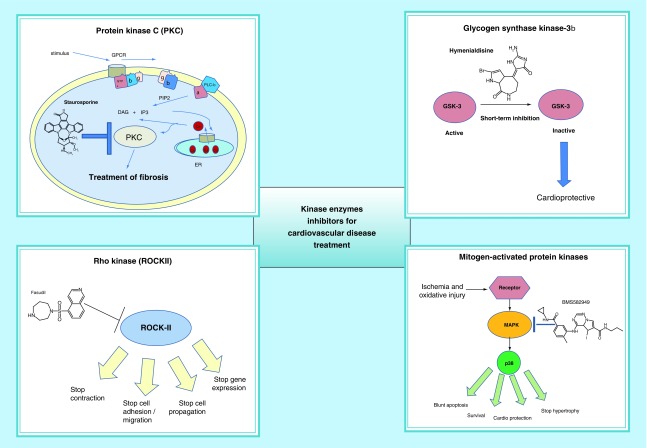

Nowadays, heart disease is being considered as the leading cause of death among men and women worldwide [1]. According to the CDC, about 610,000 people die of heart disease in the USA annually [2]. Heart disease includes coronary heart disease, heart attack, congestive heart failure and congenital heart disease with coronary heart disease being the most common type; about 375,000 Americans die of heart disease annually [2,3].

Multiple signaling pathways are associated with cardiac function, some of which are beneficial and others injurious [4]. The cardiovascular disease (CVD) state is largely affected by the balance between these signaling pathways, and selective protein kinase inhibitors are being enormously studied as potential new selective therapeutic agents that can substitute traditional receptor blockers for treating CVD [4]. For example, myocardial ischemia is claimed to be caused by the disruption of tightly structured and consistent signaling pathways. Recent studies have uncovered three emerging mechanisms of cell death in the ischemic heart [5]: first, the mitochondrial permeability transition and its effector, the mitochondrial permeability transition pore, which is regulated by the PI3K–AKT–GSK3 kinase axis; second, the programmed myocardial cell death (necrosis), which is regulated by the interacting protein 1 and 3 kinases. Finally, there is the Ca^2+^overload-induced mitochondrial dysfunction, which is regulated by mitochondrial calcium uniporter and Ca^+2^ calmodulin (CaM)-CaMK-II. Inhibition of each of these kinase pathways has been proposed as a means to limit myocyte death from ischemia/reperfusion injury [5].

Furthermore, a number of effective small molecule protein kinase inhibitors have been approved by the US FDA; the first was imatinib in 2001 [6]. Afterward, several other inhibitors were subsequently approved. Most of these FDA-approved kinase inhibitors are indicated for cancer treatment, but recently some other indications have been added to the FDA-approved drug list. For example, tofacitinib, a JAK 3 inhibitor, was approved for treatment of rheumatoid arthritis in 2012 [6]. Unfortunately, until now no kinase inhibitor drug is indicated as a safe and effective therapeutic agent for heart disease, although many pharmaceutical research efforts have been made toward this goal.

Aberrant kinase activity in CVD is due to enhanced stimulation by the activated neurohormonal system [4]. Various kinases are involved in modulating cardiac function via phosphorylation. Figure 1 summarizes the most important kinase targets that have been previously reported as kinase targets for heart disease treatment [6]. The most important are ROCK II [7,8], Ca^+2^ CaMK IIδ [9–11], protein kinase C (PKC) [12,13], PI3K [14], GSK-3β, FAK and MAPKs [15]. Various kinase inhibitors have been used as research tools to test the putative anticardiac disease activity, but unfortunately none of the lead protein kinase inhibitors has been approved as a therapeutic drug for heart disease treatment up to now.

**Figure 1.**
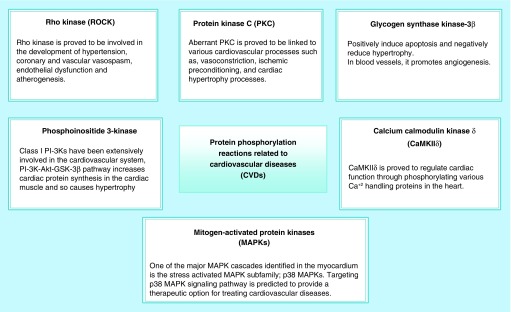
**The most important kinase targets that have been previously reported as kinase targets for heart disease treatment.** CVD: Cardiovascular disease.

This review aims toward highlighting the efforts and strategies in literature related to treating heart disease with small molecule protein kinase modulators and providing broad overview of the medicinal chemistry approaches currently used for this aim. In addition, we intend to summarize contemporary (1970–2016) reports of small molecule inhibitors claimed against various CVD-related kinases or reported to carry CVD-related kinases inhibitory activities, and categorize these compounds according to their origin. Furthermore, we intend to discuss the challenges facing the efforts exerted toward finding effective small molecule protein kinase inhibitors and still subduing them from finding new kinase drug inhibitors that can be approved for use in heart disease treatment.

## Methods

In our search, we based our kinase target selection on a review article written by Kumar *et al.* from the Cardiovascular Research Institute, College of Medicine (TX, USA) in 2007 [4]. Kumar *et al.* clearly verified Ca^2+^-CaMK-II, PKC, ROCK, PI3K and MAPK as the most important kinase targets that can attenuate the cardiac function.

After selecting the targets, we made an extensive search to identify published medicinal chemistry studies for each target from 1970 until 2016. Our search strategy involved the use of Boolean connectors for combination of terms such as ‘name of the target’, ‘inhibitor’, ‘cardiovascular’, ‘kinase’, ‘FDA’, ‘studies’, ‘pharmaceutical companies’, ‘clinical studies’ and ‘drugs’. Equivalent terms were also used whenever possible. The search was limited to full-text articles published in English language from 1970 to 2016. Studies that were written by groups of scientists working in pharmaceutical companies were extensively followed. Electronic database search included PubMed, ISI Web of Knowledge, SciFinder, Science Direct, Springer and Google Scholar. The reference lists in all retrieved article were inspected for additional information. Studies that were connecting the selected targets to disease states other than CVD were excluded.

## CaMK-IIδ

CaMK-IIδ is a predominant calcium calcium calmodulin serine/threonine kinase isoform in the heart. Many studies have established its role as an important regulator in cardiac function by phosphorylating various Ca^+2^ handling proteins in the myocardium such as phospholamban, Ryanodine receptor, L-type Ca^+2^ channel and other myofilament proteins [16–19]. And so, it is the overactivation of CAMK-II receptor that directly leads to increased cardiac muscle contraction and increased diastolic chamber stiffness, which are important factors in the pathophysiology of a range of cardiac diseases [17].

Myosin light-chain kinase (MLCK) is a family of Ca^+2^/CaM-dependent protein kinases that phosphorylate the regulatory MLC (MLC2). MLC post-translational modification is a key molecular cascade that regulates endothelial permeability and barrier function. MLCK mediated phosphorylation of ATP-dependent actomyosin contraction which increases capillary permeability. Similar to smooth muscle, in vascular endothelium, MLC phosphorylation triggers contraction, resulting in endothelial cell membrane retraction, intercellular.

MLC has several isoforms, smooth muscle and nonmuscle MLCK isoforms, respectively; they have wide tissue distribution, and both are expressed in microvascular endothelial cells. Its structure includes actin-binding, catalytic, inhibitory, CaM-binding and kinase-related protein domains, and it also contains a unique fragment containing multiple sites for protein–protein interaction as well as potential regulatory phosphorylation sites for important kinases such as PKC, protein kinase A (PKA) and MAPKs’ gap formation, and barrier compromise [20].

### Natural & semisynthetic CaMK-II inhibitors

In our search, we did not find many reports of natural CaMK-II inhibitors; one report by Mayadevi *et al.* published in 2012 [21] mentioned that curcumin, commonly named as turmeric, which is the principal curcuminoid of turmeric (*Curcuma longa*, family Zingiberaceae) has an inhibitory action on CaMK-II (compound **1**, Table 1). Also, in this same study, the scientists reported the development of semisynthetic heterocyclic analog of curcumin (3,5-bis[b-(4-hydroxy-3-methoxyphenyl ethenyl]pyrazole), named as pyrazole-curcumin, as a potent inhibitor of CaMK-II (IC_50_ = 1.49 µM) (compound **2**, Table 1) [22].

**Table 1.** **Calcium calmodulin serine/threonine kinase IIδ inhibitors in cardiac muscle.**

**No.**	**Structure**	**Inhibitor class**	**Study year**	**Activity IC_50_**
1		Natural	2012	112 µM

2		Semisynthetic	2012	1.49 µM

3		Synthetic	1990	500 nM

4		Synthetic	1996	1.57 µM

5		Group of aryl indolyl–maleimide scaffold, synthetic inhibitors	2008	10 nM to >20 µM

6		Most active aryl indolyl–inhibitor against CaMK IIδ	2008	10 nM

7		Synthetic	2012	12 nM

8		Homology modeling synthesis of pyrimidine-based inhibitors	2008	0.009–3 µM

9		Most active pyrimidine-based inhibitor	2008	9 nM

10		Synthetic	2012	2 nM

11		Inhibitor resulted from ligand-based virtual screening CaMK IIδ inhibitor	2011	20 nM

12		Inhibitor resulted from ligand-based virtual screening CaMK IIδ inhibitor	2011	82 nM

CaMK-IIδ: Calcium calmodulin serine/threonine kinase IIδ.

### Synthetic inhibitors of CaMK-II (isoquinoline sulfonamide scaffold)

CaMK-II was one of the earliest kinase targets studied to treat CVDs. In 1990, a group of Japanese scientists lead by Tokumitsu reported one of the first articles describing the synthesis of CAMK-II inhibitors [23]. It specifically described the synthesis of KN-62 (Table 1), 1-[*N*,O-bis(5-isoquinolinesulfonyl)-*N*-methyl-L-tyrosyl]-4-phenylpiperazine as a specific inhibitor of Ca^2+^/CaMK II [23].

KN-62 (Compound **3**, Table 1) was found to inhibit the CaMK-II by interacting with the CaM-binding site on the enzyme [23,24], KN-62 (compound **3**, Table 1) inhibits CAMK-II with an IC_50_ value of 500 nM [23,24] which is tenfold less potent inhibitor for GSK-3β and PKA. Furthermore, KN-62 is reported to inhibit CAMK-I and CAMK-IV at similar concentrations to CAMK-II, which renders it unsuitable to treat specific CVDs. Many synthetic analogs of KN-62 were synthesized and used as standard inhibitors for CaMK-II research studies, and many of its analogs were synthesized.

In 1996, Yokokura *et al.* reported the synthesis of a nonisoquinoline derivative of KN-62, named as HMN-709 (2-[*N*-(2-aminoethyl)-*N*-(4-chlorobenzenesulfonyl)]amino-*N*-(4-fluorocinnamyl)-*N*-methylbenzylamine) [25] (compound **4**, Table 1), with an IC_50_ value of 1.57 µM. HMN-709 was described as a CaM antagonist and classified as an ‘ATP noncompetitive’ or ‘allosteric’ compound that binds outside the active site of CAMK [25].

### Synthetic inhibitors of CaMK-IIδ (aryl–indolyl maleimide-based scaffold)

The clear role of CaMK-IIδ in modulating heart function has attracted scientists working in Scios, Inc. (a biopharmaceutical company acquired by Johnson & Johnson, USA) toward designing and developing potentially active anti-CaMK-IIδ lead inhibitors, among these were Levi *et al.* [10–11,26]. Levi *et al.* also synthesized another aryl–indolyl maleimide series of anti-CaMK-IIδ compounds with activities ranging from 10 nM to >20 µM (see scaffold **5**, Table 1) this effort was based upon manipulating aryl group and the tether joining the basic amine to the indolyl maleimide core of CaMK-IIδ inhibitors (see scaffold 5, Table 1). The most active compound in this series of inhibitors was compound **6** in Table 1 with nanomolar activity (IC_50_ = 10 nM).

Furthermore, in 2012 Dainippon Sumitomo Pharma^®^ in Osaka, Japan reported the synthesis and of 2-(4-phenoxybenzoyl)-5-hydroxyindole as a novel series of CaMK-II kinase inhibitors. The most potent inhibition of CaMK-II was seen with the dibromo compound Dainippon-25 (IC_50_ = 12 nM) (compound **7**, Table 1) [27].

### Homology modeling & synthesis of pyrimidine-based inhibitors of CaMK-IIδ

In 2008, Mavunkel *et al.* (Scios, Inc.) built a homology model of CaMK-IIδ based on the crystal structure of autoinhibited rat CAMKI (Protein Data Bank code: 1A06) and used the resulted model to synthesize new series of non-ATP competitive pyrimidine based CaMK-IIδ inhibitors [11]. The resulted compounds exhibited an IC_50_ value ranging from 0.009 to 3 µM (see scaffold **8** and compound **9**, Table 1).

Later on, in 2012, Beauverger *et al*. registered a patency for Sanofi^®^ (Paris, France) describing the synthesis of 5-oxo-5,8-dihydropyrido[2,3-d]pyrimidine derivatives as CAMK-II kinase inhibitors for treating CVDs [28], Patency No. US 20120277220 A1. The most active compound is Sanofi-32, IC_50_ = 2 nM (compound **10**, Table 1) [28].

### Ligand-based drug design of CaMK-IIδ inhibitors

Virtual screening methodologies are broadly used nowadays to find novel specific kinase inhibitors. Specifically, ligand-based 3D pharmacophores were developed for CaMK-IIδ by Shahin and Taha in 2011 [29]. This study embraced the use of multiple linear regressions and genetic function algorithm in order to build a predictive quantitative structure–activity relationship equation that can describe the activity of potent CaMK-IIδ inhibitors [30]. Finally, this approach resulted in several active inhibitors ranging in their activity from 0.02 to 2.46 µM (see compounds **11** and **12**, Table 1) [29].

### What is in the future for CaMK-IIδ as a target to treat CVD?

By tracing the development of CAMK-II inhibitors to treat CVD from 1990 till date, we can observe tremendous efforts toward finding new inhibitors for this target. This is due to the well-established scientific certainty about the important role of CaMK-IIδ in the development of heart pathologies [28,31]. Unfortunately, the selectivity has been always an issue in this area. Compounds that target the allosteric binding pocket of CaMK-II are very promising, but still more scientific effort should be made to reveal the secrets of the allosteric binding site of CAMK-II and to design new inhibitors that can target mutually the hinge region of the ATP-binding pocket and the allosteric site of CAMK-II; this strategy will help in potentiating both the potency and the selectivity of the newly synthesized inhibitors since the sequence homology among the allosteric sites of different kinase inhibitors is quite low, and this provides new opportunities for more specific and minimal off-target inhibition [32,33].

## ROCK

ROCK is a kinase enzyme that belongs to the family of serine/threonine kinase enzymes. ROCK mainly regulates the movement of cells by organizing the actin cytoskeleton, cell adhesion and motility [34]. There are two isoforms of ROCK (ROCK-I and ROCK-II); ROCK-II is preferentially expressed in heart muscle tissue and aberrant ROCK activity has been also linked to various cardiac physiological processes, such as cardiac myocyte hypertrophy, ion channel activity, endothelial permeability and reactive oxygen species production [4,35].

The successful revelation of ROCK’s pathophysiological role in heart disease has established its role as a key target for treating CVDs. In addition, the validity of ROCK-II as a target for therapeutic intervention was established by emerging clinical trials [34].

### High-throughput screening trials searching for ROCK inhibitors

A high-throughput screening search campaign aimed toward finding new active Rock inhibitors was conducted by Schroter *et al.* in 2008 [36]. Throughout this campaign, an institutional library consisting of approximately 600,000 substances was screened for ROCK inhibitors. Finally, this led to the discovery of new pyridine–thiazole-based selective ROCK-II inhibitors, and the most potent was of an IC_50_ value of 7.2 nM (see compound **13**, Table 2) [36,37].

**Table 2.** **Rho kinase inhibitors.**

**No.**	**Structure**	**Inhibitor class**	**Publication year**	**Activity**
13		HTS compound	2008	IC_50_ = 7.2 nM

14		Synthetic ‘isoquinoline prototype’	1997	K_i_ = 330 nM

15		Fasudil derivative approved for glaucoma treatment in Japan	2014	ROCK-I (IC_50_ = 51 nM); ROCK-II (IC_50_ = 19 nM)

16		Synthetic ‘isoquinoline series’	1997	K_i_ = 1.6 nM

17		Synthetic ‘4-aminopyridine series’	1997	*K*_i_ = 0.14 µM

18		Synthetic ‘pyrrolopyridine series’	2007	ROCK-II (IC_50_ = 3.6 nM)

19		Synthetic ‘indazole series’	2006	IC_50_ = 13 nM

20		Synthetic benzadioxane-based compound	2008	ROCK-I (IC_50_ = 56 nM)

21		Tetrahydroisoquinoline-based inhibitor	2010	ROCK-II (IC_50_ <1 nM)

22		Selective ROCK-II inhibitor	2011	ROCK-II (IC_50_ = 1.7 nM)

23		Tricyclic pyridocarboxamide derivatives	2015	ROCK-II (IC_50_ = 1 nM)

24		Structure-based drug design inhibitor	2014	IC_50_ = 1.5 μM

25		Structure-based design inhibitor	2013	ROCK-II (IC_50_ = 0.02 μM)

26		Ligand-based drug design inhibitor	2011	ROCK-II (IC_50_ = 0.02 μM)

HTS: High-throughput screening; ROCK: Rho kinase.

### Synthetic ROCK inhibitors

A wide range of synthetic ROCK inhibitors were synthesized and studied in the last two decades. The first was a low-molecular-weight compound, named as fasudil (K_i_ = 330 nM) against ROCK-II (see compound **14**, Table 2) [38]. Fasudil was described in 1997 in a paper published by a Japanese group of scientists, Uehata *et al.* [39]. fasudil (an isoquinoline derivative) inhibits ROCK via targeting ATP-binding domain in the ROCK kinase, and it has been reported as an effective ROCK inhibitor for treating a wide range of CVDs in clinical studies such as coronary vasospasm, angina, hypertension and heart failure [36,37]. However, it has been reported to have only moderate kinase selectivity [40]. Nevertheless, fasudil was approved in 1995 as a drug for cerebral vasospasm treatment in Japan (30 mg iv. three-times a day for 14 days) [41,42].

In 2014, ripasudil (a fasudil synthetic derivative, also known as K-115; see compound **15**, Table 2) was approved for the treatment of glaucoma in Japan [40,43–45]. Presently, fasudil is undergoing clinical trials for the treatment of ischemic heart disease [33].

Chemical optimization of Fasudil also led to another isoquinoline derivatives, for example, compound H-1152 (K_i_ = 1.6 nM; compound **16**, Table 2). H-1152 is a compound that exemplifies an excellent investigational tool for ROCK inhibition *in vitro* [40,46–47] but yet it is not approved for treatment of any medical condition.

The efforts to develop selective analogs of Fasudil continued. In 2012, Lavogina *et al.* reported the synthesis of conjugates between the 5-isoquinolinesulfonylamide fasudil and the oligo-d-arginine. This compound possesses over 160-fold selectivity toward ROCK compared with that against PKA [48].

Later on, several other reports revealed the synthesis of new ROCK inhibitors based on other chemical classes; for example, the 4-aminopyridine, indazole, amide and urea series.

The most potent compound of 4-aminopyridine series is Y-27632 (K_i_ = 0.14 µM, compound **17**, Table 2). This compound was presented as an effective potential therapeutic agent to decrease ischemia–reperfusion injury and myocardial fibrosis after myocardial infarction (MI), and in a rat model for chronic hypertension [4,49]. The other analogs of 4-aminopyridine ROCK-II inhibitors were synthesized but still inferior in their potencies – for example, compound Y32885 (K_i_ = 0.2 µM) [40]. The 4-aminopyridine ROCK-II inhibitors were chemically modified to the pyrrolopyridine series and again the selectivity was relatively moderate in these compounds although the potency was modified, for example, compound Y39983 (IC_50_ vs ROCK-II is 3.6 nM; compound 18, Table 2) [50].

The well-established validity of ROCK as a target for therapeutic intervention in treating heart diseases and other diseases encouraged the scientist to continue researches on finding new and selective ROCK-II inhibitors. Extensive synthetic studies were conducted in this area; for example, many compounds such as methylenephenyl, benzimidazole, benzothiazole and indazole-substituted pyrazole derivatives were synthesized and studied as ROCK inhibitors.

Indazole-based series of compounds were synthesized by Feng *et al.* in 2007. Compound SR-1459 (compound **19**, Table 2) was the most potent compound in the indazole series at that time and its IC_50_ versus ROCK-II is 13 nM [51].

Then in 2008, the same group of scientists presented a new potent benzadioxane-based compound named as SR-3677 (compound **20**, Table 2) with an IC_50_ value of 3 nM against ROCK-II, an IC_50_ value of 56 nM against ROCK-I and an IC_50_ value of 3970 nM against PKA, and this was considered a breakthrough toward finding selective inhibitors for ROCK-II enzyme [40,52]. Again, in 2010 Feng *et al.* reported the synthesis of new tetrahydroisoquinoline derivatives as ROCK inhibitors. These compounds were characterized by their high potency, high selectivity and appropriate pharmacokinetic properties. The most potent compound in this series possessed a nanomolar IC_50_ value (IC_50_ < 1 nM; compound **21**, Table 2) [40,53]. In 2011, Feng *et al.* modified SR-3677 (compound **20**, Table 2) by adding a substituted bulky quinazolinone core; this approach lowered the PKA inhibition of the newly synthesized compounds and increased their selectivity against ROCK-II enzymes, for example, compound SR-6246 exhibited an IC_50_ value of 1.7 nM against ROCK-II, an IC_50_ value of 6.7 nM against ROCK-I and an IC_50_ value of more than 14 µM against PKA (see compound **22**, Table 2) [40].

In 2015, Bristol-Myers Squibb (BMS; NY, USA) registered a patency for tricyclic pyridocarboxamide derivatives as inhibitors against the ROCK enzyme, and the most potent compound in this patency exhibited an IC_50_ value of <1 nM for ROCK-II (see compound **23**, Table 2) [54].

### Structure-based drug design of ROCK-II inhibitors

A structure-based study of ROCK inhibitors was first published in 2010 by Mishra *et al.* [55]. This study was based on a structure-guided design of eight co-crystal structures of ROCK–inhibitor complexes. Then, the energy-based pharmacophores resulted from this study were used as filters to virtually screen the commercially available Asinex database containing 525,807 molecules. This study resulted in identifying new ROCK inhibitors. The most potent compound in this study showed an IC_50_ value of 1.5 μM against ROCK kinase (compound **24**, Table 2). The low micromolar activity of the most active resulted compound in this study prompted the authors to mention that the identified lead may constitute a prototypical molecule for further optimization as an anti-ROCK inhibitor [55].

In 2013, a group of Korean scientists reported the discovery of new potent ROCK inhibitor named as DW1865 (compound **25**, Table 2, IC_50_ against ROCK-II = 0.02 μM) [56] by applying rational virtual discovery strategies including structure-based drug design and straight structure–activity relationship. DW1865 was described to be ten-times more potent in inhibiting ROCK activity than Fasudil. In addition, the activity of DW1865 was shown to be highly selective for ROCK-II compared with 13 other kinases. DW1865 was described to cause vasorelaxation in phenylephrine- or 5-hydroxytriptamine-induced contraction in a concentration-dependent manner and to exert a significant and dose-related reduction in blood pressure in spontaneously hypertensive rats [56].

### Ligand-based drug design of ROCK-II

Ligand-based 3D pharmacophores were developed for ROCK by Shahin and Taha in 2011 [57]. This work included multiple linear regressions and genetic function algorithm processes in which several 2D and 3D descriptors were allowed to compete for building a predictive quantitative structure–activity relationship model. Eight submicromolar ROCK-II inhibitors were identified. The most potent ones have IC_50_ values of 0.7 and 1.0 µM [57] (most active is compound **26**, Table 2).

### Current status of ROCK-II as cardiovascular treatment target

As we have described earlier, extensive studies were applied by several research groups to identify novel, potent and selective inhibitors against ROCK-II; from our point of view, these studies were not targeted against ROCK-II as the key role player in the pathogenesis of heart disease. Instead, these studies emphasized on ROCK-II as a target to treat several other diseases such as cerebral vasospasm, glaucoma, diabetic retinopathy, kidney disease and even psoriasis [58,59]. Although there were major advances in finding ROCK inhibitors to treat these diseases, for example, compound **Ar-13324** (structure not disclosed) developed by Aries^®^ has reached Phase II in clinical studies to treat glaucoma [58,59] but yet no ROCK inhibitors were registered to treat any CVD [58,59].

Based on the fact that many ROCK-II inhibitors were identified to be potent and selective in addition to the fact that the role of ROCK-II in the pathogenesis of heart disease is very well established, we suggest that the next step should emphasize on bridging the gap between the previously synthesized ROCK-II inhibitors and their efficacy in treating hypertensive and heart-related conditions in animal models and clinical studies.

## Protein kinase C

PKC enzymes are group serine/threonine kinases that are expressed in many tissues inside the human body, including the myocardium tissue [60]. It has been recently cited that some of PKC isozymes are implicated in the propagation of different types of heart diseases; for example, PKC-α activation was correlated with decreased cardiac contractility and uncoupling of β-adrenergic receptors [61–63]. Also, PKC-βII was shown to be involved in dysregulation of calcium handling in myocardium tissue [64,65]. In addition to that, activation of PKC-ϵ in heart muscles was accompanied with increased fibrosis, fibroblast proliferation and inflammation which may finally lead to heart failure [66].

### Natural inhibitors of PKC

The indolocarbazole alkaloid staurosporine was first isolated by Omura *et al.* from *Streptomyces staurosporeus* in 1977 [67]. Later on in 1986, the inhibitory activity of Staurosporine against PKC was revealed (IC_50_ = 2.7 nM) [67,68]. This discovery opened the door widely toward new drug discovery trials targeting kinases in general. Staurosporine (compound **27**, Table 3) was first named as AM-2282; later on, many of its analogs were synthesized. Another series of PKC inhibitors was isolated from the culture of *Nocardiopsis* species; among them are K-252a (IC_50_ = 18–25 nM; compound **28**, Table 3), K-252b (IC_50_ = 20 nM; compound **29**, Table 3) and K-252c (IC_50_ = 2.45 µM) [68–70]. Furthermore, in 1992, RK-1409 (7-oxo Staurosporine; compound **30**, Table 3) was isolated from *Streptomyces platensis*, subsp. *Malvinus* and showed significant PKC inhibitory activity (IC_50_ ≈ 1 nM) [68–70]. Several other Staurosporine derivatives were reported to be isolated and reported in the literature but all with submicromolar PKC inhibitory concentrations [68–70].

**Table 3.** **Protein kinase C inhibitors.**

**No.**	**Structure**	**Inhibitor class**	**Publication year**	**Activity**
27		Natural inhibitor (Staurosporine)	1977, first isolated; 1986, the inhibitory activity against PKC was revealed	PKC (IC_50_ = 2.7 nM)

28		Semisynthetic (K-252 a)	1986	IC_50_ = 18–25 nM

29		Semisynthetic (K-252b)	1986	IC_50_ = 20 nM

30		Semisynthetic, 7-oxo Staurosporine (RK –1409)	1992	IC_50_ ≈ 1 nM

31		Natural (Calphostin C)	1989	IC_50_ = 0.05 µM

32		Synthetic (AEB071)	2009	PKC-α (IC_50_ = 2.1 nM) PKC-ϵ (IC_50_ = 6.1 nM)

33		Synthetic	2011	PKC-α (IC_50_ = 829 nM) PKC-ϵ (IC_50_ = 5 nM)

34		Synthetic	2011	PKC-βII (K_i_ = 29 nM)

35		Flosequinan-selective PKC inhibitor	1992	–

36		Ruboxistaurin	2006	PKC-βI (IC_50_ = 4.7 nM) PKC-βII (IC_50_ = 5.9 nM)

PKC: Protein kinase C

Furthermore, Other PKC natural inhibitors named as calphostins A–E were reported in the literature. These compounds were isolated from the fungus *Cladosporium cladosporioides* (IC_50_ ranging from 0.05 to 6.36 µM) with Calphostin C (UCN-1028C) showing the highest potency among them (compound **31**, Table 3) [71].

### Synthetic inhibitors of PKC

In 2009, Wagner *et al.* synthesized new maleimide-based series of compounds and evaluated them against different types of PKC isotypes [72]. In this study, compound AEB071 (compound **32**, Table 3) was the most potent with activities against PKC-α (IC_50_ = 2.1 nM), PKC-ϵ (IC_50_ = 6.1 nM) [72]. Later, in 2011 Van Eis *et al.* reported the synthesis of new 2,6-naphthyridine derivatives as PKC inhibitors; compound **11** in the reported paper (compound **33**, Table 3) was the most potent PKC-α (IC_50_ = 829 nM), PKC-ϵ (IC_50_ = 5 nM) [73]. Another study was published in 2011 by Li *et al.* which identified novel pyrrolopyrazoles series of PKC-βII inhibitors [74]; compound **23** in the reported paper showed superior results (K_i_ = 29 nM) against PKC-βII (see compound **34**, Table 3).

### Clinical cardiovascular trials related to PKC inhibition

The drug flosequinan (see compound **35**, Table 3) is a nonselective PKC inhibitor that was indicated for treatment of congestive heart failure. It was sold under the name Manoplax^®^ and then was withdrawn from the US market in October 1993 due to increased hospitalization and mortality [75,76]. Also, ruboxistaurin (LY333531; see compound **36**, Table 3) is a selective inhibitor of different PKC isoforms; PKC-β1 and PKC-β2 (IC_50_ = 4.7 and 5.9 nM, respectively) [75,76]. Moreover, the IC_50_ values for PKC isoforms α, γ, δ, ϵ, ζ and η are 360, 300, 250, 600, >100,000 and 52 nM, respectively [75,76]. Ruboxistaurin (LY333531) was introduced by Elli lilly as an investigational drug for treatment of diabetic peripheral neuropathy and retinopathy. Unfortunately, no progress was informed for this proposal. On May 2006, Elli lilly filed with Europe, the Middle East and Africa. Its current development status in the Europe is unclear at this stage, but still investigational clinical studies for this indication are ongoing [75]. Finally, on 15 March 2007, Eli Lilly withdrew its marketing authorization application for ruboxistaurin for diabetic retinopathy and from the FDA [77].

### PKC as a future cardiovascular treatment target

The exact role of different isoforms of PKC in cardiac disease is still ambiguous; taking into consideration that the PKC family of enzymes includes three classes and 12 isoforms, and that PKC isoforms have four structurally conserved domains (C1–C4) and five variable domains (V1–V5) [78] accordingly we can say that the selectivity of the PKC targeting drugs is an issue that can cause unacceptable on-target and off-target effects [79]. The future PKC targeting drugs should be more focused on monitoring the pharmacodynamics of new PKC modulators and revealing more information about the biology of various PKC isoforms.

## p38 MAPK

MAPKs include many serine–threonine kinases; one of the major MAPK cascades identified in the myocardium is the stress-activated MAPK subfamily; p38 MAPKs [80]. Targeting p38 MAPK signaling pathway is established to provide a therapeutic option for treating CVDs [80,81]. For example, it has been proven that myocardium p38 MAPK was evidently activated throughout the development of cardiac hypertrophy disease and inactivated during decompensation stages [80,82]. In addition, p38 MAPK inhibition has been demonstrated to blunt apoptosis *in vivo* and *ex vivo* cardiac ischemia/reperfusion injury models [83]. Also, cardio protection is also observed in a transgenic heart expressing a dominant negative mutant of p38α MAPK [83]. All of these studies and many others encouraged the scientists in pharmaceutical companies to search for new p38 MAPK active hits.

### Natural inhibitors of p38 MAPKs

Naturally occurring polyphenols are antioxidants that are able to attenuate MAPK-induced signaling pathways, and so phenolic compounds are able to decrease inflammatory reactions, platelet aggregation processes, atherosclerosis and hypertension thus protecting the cardiac muscles [84].

Phenolic compounds cover a wide range of natural compounds; the most important group is the flavonoids such as epigallocatechin gallate (found in tea). Epigallocatechin gallate has been found to attenuate angiotensin-II and pressure overload mediated cardiac hypertrophy [80,84]. Quercetin is also a flavonoid polyphenol, and it is also found to abolish hydrogen peroxide-induced endothelial cell apoptosis via blocking the phosphorylation of p38 MAPK [80].

Moreover, resveratrol is a type of natural phenol found in grapes, blueberries, raspberries and mulberries, and is also proposed to treat ischemic heart disease via attenuating MAPK/SAPK/cAMP response element-binding protein [80].

Furthermore, all of the previously mentioned natural inhibitors of p38 MAPKs were based on preclinical researches and yet no actual natural inhibitor of p38 MAPKs is considered as a real drug to treat stress related-cardiac hypertrophy or any cardiac condition.

### Synthetic inhibitors of p38 MAPKs

One of the first synthesized compounds against p38 MAPKs is the SmithKline Beecham compound SB 203580 (compound **37**, Table 4) [85]. SB 203580 was first synthesized in 1996 and reported to inhibit p38 MAPK, nitric oxide production and inducible nitric oxide synthase in bovine cartilage-derived chondrocytes; nowadays it is considered as the literature standard for p38 kinase inhibitor (IC_50_ = 0.3–0.5 μM) [86].

**Table 4.** **MAPK inhibitors.**

**No.**	**Structure**	**Inhibitor class**	**Publication year**	**Activity**
37		Synthetic (SB 203580)	1996	IC_50_ = 0.3–0.5 µM

38		Synthetic (SB 220025)	1998	IC_50_ = 60 nM

39		Synthetic (RWJ 67657)	1999	p38α (IC_50_ = 1 μM) p38β (IC_50_ = 11 μM)

40		Synthetic (VX-702)	2007	p38α (IC_50_ = 4–20 nM)

41		Synthetic (Scio-469)	2006	p38α (IC_50_ = 9 nM)

42		Synthetic Dilmapimod (SB681323)	2005	Under study in early phase

43		Synthetic	2007	IC_50_ = 3 nM

44		Synthetic	2007	IC_50_ = 3 nM

45		BMS582949	2006–2015	p38α (IC_50_ = 13 nM)

46		Synthetic (VX-745)	1999	p38α (IC_50_ = 5 nM)

Moreover, in 1998 SmithKline Beecham synthesized compound SB 220025 (compound **38**, Table 4), which is also a specific inhibitor of human p38 MAPK with an IC_50_ value of 60 nM [86].

In 1999, Johnson Pharmaceutical Research Institute reported the synthesis of RWJ 67657 (compound **39**, Table 4) which is selective p38α and p38β inhibitor (IC_50_ = 1 and 11 μM, respectively). RWJ 67657 displayed no activity at p38γ and p38δ, and was reported to exhibit cardio protective and anti-inflammatory activity in addition to being orally active [87]. RWJ 67657 is approximately tenfold more potent than the literature standard of p38 kinase inhibitor SB 203580 (compound **37**, Table 4) in all p38-dependent *in vitro* systems tested [87].

Again, scientists were interested in p38 MAPK as a target to treat chronic inflammatory diseases such as rheumatoid arthritis. Nevertheless, some synthesized p38 MAPK inhibitors were indicated to treat congestive heart failure such as VX-702 (compound **40**, Table 4), Scio-469 (compound **41**, Table 4) and Dilmapimod (SB681323; GlaxoSmithKline) (compound **42**, Table 4). Dilmapimod (SB681323) is still under study in early phase (Phase IIa) as a treatment for acute lung injury and acute respiratory distress syndrome to evaluate its safety and tolerability, as a potent, selective inhibitor of p38α (MAPK) [88]. Unfortunately, SB681323 is not filed to treat stress-related congestive heart failure though it was one of its proposed indications in the early drug design stages [88].

In the following years, many groups of scientists continued the search for new inhibitors to p38 MAPK; for example, a German group led by Laufer *et al*. from the Institute of Pharmacy, Eberhard-Karls-Uni**v**ersity reported the synthesis of novel tri- and tetra-substituted imidazole analogs as inhibitors of p38 MAPK [89], with most active compound **43**, Table 4 (IC_50_ = 3 nM). Simultaneously, a group of scientists from the USA led by Das from BMS reported the synthesis pyrazolopyrimidine-based series of compounds as a novel scaffold for selective p38a inhibitors. In this study, the most active compound was compound **44** (Table 4; IC_50_ = 3 nM) [89, 90]. In 2015, BMS filed compound BMS582949 (see compound **45**, Table 4) for Phase II clinical trial as a treatment for atherosclerosis [91]. Unfortunately, the finding of the study revealed that treatment with BMS582949 did not reduce arterial inflammation relative to placebo compared with statins [91], but compound BMS582949 is still under study to treat moderate-to-severe plaque psoriasis [92].

The efforts are going on to synthesize new p38 MAPK inhibitors to treat cardiac myopathies but, as we mentioned, other than compound BMS582949, no compound has been filed to the FDA for this purpose. Hopefully later on, research data will reveal more about p38 inhibitors that have therapeutic use beyond their well-known anti-inflammatory properties as stress-related cardiac hypertrophy drugs [80].

### MAPK as a cardiovascular treatment target

Noticeably, no active p38 MAPK inhibitor for treating stress-related cardiac hypertrophy has reached the market. Yet, some p38 MAPK inhibitors such as Vertex 745 (VX-745) (compound **46**, Table 4) and SCIO-469 (compound **41**, Table 4) are considered as pharmacological p38 MAPK inhibitors that are under development for treating rheumatoid arthritis [93–95]. In the previous discussion, we discussed about some of the efforts to find new p38 MAPK inhibitors to treat stress-related cardiac hypertrophy. We can see that the pharmaceutical companies are interested in finding MAPK inhibitors to treat the inflammatory condition associated with the atherosclerosis pathogenesis, but no MAPK inhibitor has proved to be superior over statins in this regard. As for p38 MAPK, it is crystal clear that we are a few steps away from finding a new kinase inhibitor to treat CVD hopefully in the near future; we will see such a drug in the market.

## GSK-3β

In myocardium, GSK-3β positively regulates apoptosis and negatively regulates hypertrophy [4,96]. Inhibition of GSK-3β has been proposed as an approach to improve postischemic cardiac myocyte survival and enhance cardioprotection [4,97]. In addition to that, it was found that short-term inhibition of GSK may be cardioprotective; however, long-term or sustained inhibition of GSK-3β may contribute to the development of hypertrophy.

### Natural inhibitors of GSK-3β

In a screening study that was conducted and published by Meijer *et al.* in year 2000, the marine sponge constituent hymenialdisine (compound **47**, Table 5) was found to be a potent inhibitor of GSK-3β (IC_50_ = 0.01 µM) [98]. In 2001, another study conducted by Leclerc *et al.* revealed the discovery of indirubin as a new natural inhibitor of GSK-3β [99]. Indirubin was extracted from Danggui Longhui Wan, a traditional Chinese medicine, and was found to potently inhibit GSK-3β (IC_50_ = 0.009 nM; compound **48**, Table 5) [99].

**Table 5.** **Inhibitors of glycogen synthase kinase-3β.**

**No.**	**Structure**	**Inhibitor class**	**Publication year**	**Activity**
47		Natural (hymenialdisine)	2000	IC_50_ = 0.01 µM

48		Natural (5-iodo-indirubin-3'-monoxime)	2001	IC_50_ = 0.009 nM

49		Synthetic (alesterpaullone)	2000	IC_50_ = 0.004 nM

50		Synthetic (aloisine A)	2002	IC_50_ = 0.65 µM

51		Synthetic	2003	IC_50_ = 0.018 µM

### Synthetic inhibitors of GSK-3β

In 2000, Leost *et al.* reported that paullones, a family of synthetic benzazepinones, act as potent inhibitors of GSK-3β (IC_50_: 4–80 µM) [100]. Table 4 shows the structure of alesterpaullone (compound **49**; IC_50_: 4 µM) [100].

In 2002, a group of scientists (Metty *et al*.) [101] performed a selectivity study on 26 kinases. This study showed that the synthetic compound aloisine A (compound **50**, Table 5) is highly selective for GSK-3β (IC_50_ = 0.65 µM), but still GSK-3β was evaluated against cancers and neurodegenerative disorders such as Alzheimer’s disease but not for heart disease treatment [101]. Later on in 2003, Zhang *et al*. synthesized a novel series of macrocyclic bisindolyl maleimides containing linkers with multiple heteroatoms and evaluated against GSK-3β (compound **51**, Table 5) [102].

Obviously, the efforts that have been made to find active inhibitors by the pharmaceutical companies against GSK-3β are much less than those made to find inhibitors against other previously mentioned targets. This might be due to the fact that we still need extra studies and researches to establish the role of GSK-3β as a useful therapeutic target in the future for treating different kinds of diseases including the CVD. In addition to that, extensive search campaigns should be supported by the pharmaceutical industry to find new inhibitors against GSK-3β.

## PI3K

PI3K has been widely investigated and validated as an anticancer target; recently, new studies are focusing on class IA isoform of PI3K as a validated target for treating heart diseases [103]. Activation of PI3K (IA) is being investigated as a new approach for the treatment of heart failure [103–105].

### PI3K natural activators

**Figure 2.**
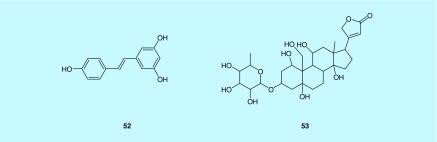
**Five PI3K/AKT/eNOS activators.** Compound **52**: resveratrol; compound **53**: the cardiac glycosides (ouabain).

Resveratrol (3,5,4′-trihydroxy-*trans*-stilbene) (Figure 2) is a natural antioxidant that derived from the skin of grapes and berries [106]. A study published in 2015 by Chong *et al.* investigated the therapeutic effect of resveratrol in arterial fibrillation prevention and tested the hypothesis that the arterial fibrillation suppression mechanism works along the PI3K/AKT/eNOS pathway [106] [107]. Finally, this study set a conclusion that resveratrol regulates ionic channels and attenuates fibrosis through activation of the PI3K/AKT/eNOS signaling pathway [106]. However, no further investigation was done by this group to assess the activity of resveratrol as PI3K natural activators.

The cardiac glycoside ouabain (compound B) was also investigated as a PI3K-IA activator by Duan *et al.* [103]. **Ouabain** is already known to protect the heart muscle against ischemia–reperfusion injury, which is usually manifested clinically as acute MI [103]. Duan *et al.* used a pharmacological approach to study the mechanism by which ouabain works as a cardioprotector and was able to conclud that PI3K-IA is actually involved in ouabain activity, but further studies are still needed to reveal the exact mechanism by which ouabain activates PI3K-IA [103].

### PI3K synthetic activators

No synthetic activators of PI3K-IA are available in the literature. We recognized that all designed ligands for PI3K are inhibitors and are intended to inhibit the p110α isoform of PI3K which is genetically linked to the occurrence of cancer but not to heart diseases [108].

## FAK

FAK is a tyrosine kinase that facilitates cytoskeletal remodeling through the localization and activation of GTPase-activating proteins (GAPs). Activated FAK enhances the activation of diverse signaling pathways important in the regulation of cell migration [109]. In addition to its role in cell-to-extracellular matrix connection, it plays a significant role in cell proliferation, migration, adhesion and survival in a wide range of cell types. FAK has recently received attention as a potential mediator of fibrosis in several cell types such as arterial and cardiac fibrosis processes. However, long-term effects on cardiac function and adverse cardiac remodeling are not clearly investigated [109]. A series of F-18 labeled 5-bromo-N2-(4-(2-fluoro-pegylated)-3,5-dimethoxyphenyl)-N4-(4-ethoxyphenyl)pyrimidine-2,4-diamine derivatives was prepared and evaluated against FAK [110], but no further inhibitors were found against this target.

## G-protein-coupled receptor kinases

G-protein-coupled receptors represent one of the largest families of membrane receptors that are responsible for regulating various biological processes. G-protein-coupled receptors are associated with heterotrimeric G-proteins and intracellular signaling pathways. G-protein-coupled receptor kinases (GRKs), along with β-arrestins, often desensitize receptor signal transduction, but the role of GRKs in cardiovascular pathophysiology is still not clear. Changes in GRK expression have been featured in many cardiovascular pathologies, including heart failure, MI, hypertension and cardiac hypertrophy. GRKs are now intensively studied as potential diagnostic or therapeutic targets.

## Conclusion

The history of protein kinase research is massively rich with many biological discoveries in the field of CVD, and significant progress has been happening in this field since the discovery of Staurosporine in 1986. Various kinase inhibitor scaffolds have been synthesized and many natural products have been explored to treat CVDs, yet none has found its way to the market as a cardiovascular disease treatment; thus, more scientific efforts are needed in this regard. The future road to find new kinase drug to treat CVD is still full of obstacles, but we hope that the near future will carry to us good news

## Future perspective

We really emphasize the importance of research efforts with an aim of finding specific and selective kinase inhibitors for treating CVDs by investing both the academic and scientific funding organizations’ efforts simultaneously in the preclinical and clinical stages of drug development.

Nevertheless, the future road to a new kinase targeting cardiovascular drug is still full of obstacles because of the high degree of conservation in the protein amino acid sequence among different kinases. As such, the implementation of computational new methods in early stages of drug design may contribute significantly to developing specific and selective kinase inhibitors based on the differences in the binding pockets’ amino acid sequences.

Protein kinases are known to be ubiquitous, meaning that kinase inhibitors could give different therapeutic results in different tissues, and that is why specificity has been always an issue regarding the design of new therapies targeting protein kinases. Academic efforts should be set toward finding dissimilarities in the amino acid sequences between the kinases that are abundant in the heart and that are in other tissues. Furthermore, these dissimilarities should be exploited to find new specific active inhibitors of the heart kinases.

In future, ongoing design of new scaffolds against kinase enzymes is encouraged, but the collaboration between academia and pharmaceutical companies will help in moving the research results from the preclinical studies toward the different stages of clinical studies and finally to the market. As has seen, much preclinical research has been published in the literature, yet a few have expanded and continued. Pharmaceutical companies should adopt these small groups of researchers from all over the world and encourage them to continue their research. This especially applies to those researchers who work on natural products in distinctive natural areas; nature is replete with phytochemical agents that can serve as a lead compound to treat different diseases.

Also, academic research should be directed toward studying new localized drug-delivery systems designed for the purpose of targeting signaling pathways that are associated with cardiac function; specifically, localized drug-delivery systems are known to decrease the toxicity issues of drugs in general.

Finally, protein kinases are proven to be activated in heart disease and their inhibition has been shown to improve cardiac function, and so the efforts to find new kinase inhibitor active drug should continue due to the well-established role of kinases in the future.

**Executive summary**

- Many kinase inhibitors have been synthesized in the last two decades, yet none has found its way to the market as a cardiovascular disease (CVD) treatment.
- Calmodulin-dependent protein kinase (CaMK)-IIδ was one of the earliest kinase targets studied to treat CVDs. Many synthetic inhibitors for this enzyme were described in the literature. The most important are KN-62 and its derivatives, aryl–indolyl maleimide-based derivatives and Dainippon-25 (IC_50_ = 12 nM).
- CaMK-II are very promising, but still more scientific effort should be made to reveal the secrets of the allosteric binding site of CAMK-II and to design new inhibitors that can target mutually the hinge region of the ATP-binding pocket and the allosteric site of CAMK-II.
- Extensive studies have been done by several research groups to identify novel, potent and selective inhibitors against Rho kinase II (ROCK-II). Major advances in finding ROCK inhibitors were achieved; for example, compound Ar-13324 (structure not disclosed) developed by Aries^®^ has reached Phase II in clinical studies to treat glaucoma but not CVDs.
- The future of protein kinase C (PKC)-targeting drugs should be more focused on monitoring the pharmacodynamics of new PKC modulators and revealing more information about the biology of different PKC isoforms.
- As for targeting p38 MAPK for CVD treatment, it is crystal clear that we are a few steps away from finding new kinase inhibitors to treat the inflammatory condition associated with atherosclerosis. Hopefully, in the near future we will see such a drug in the market.
- Extra studies and research are warranted to establish the role of glycogen synthase kinase 3β as a useful therapeutic target in the future for treating CVD.
- The future road to find new kinase drug to treat CVD is still full of obstacles, but we hope that the near future will carry to us good news.

## References

[1] Mozaffarian D, Benjamin EJ, Go AS (2014). Heart Disease and Stroke Statistics-2015 Update. A report From the American Heart Association. *Circulation*.

[2] Centers for Disease Control and Prevention Heart disease facts. http://www.cdc.gov/heartdisease/facts.htm.

[3] Xu J, Murphy SL, Kochanek KD, Bastian BA (2016). Deaths: final data for 2013. *Natl Vital Stat. Rep.*.

[4] Kumar R, Singh VP, Baker KM (2007). Kinase inhibitors for cardiovascular disease. *J. Mol. Cell Cardiol.*.

[5] Vagnozzi RJ, Hoffman NE, Elrod JW, Madesh M, Force T (2012). Protein kinase signaling at the crossroads of myocyte life and death in ischemic heart disease. *Drug Discov. Today Ther. Strateg.*.

[6] Roskoski R (2015). USFDA approved protein kinase inhibitors. http://www.brimr.org/PKI/PKIs.htm.

[7] Kolluru GK, Majumder S, Chatterjee S (2014). Rho-kinase as a therapeutic target in vascular diseases: striking nitric oxide signaling. *Nitric Oxide*.

[8] Kumar R, Singh VP, Baker KM (2007). Kinase inhibitors for cardiovascular disease. *J. Mol. Cell Cardiol.*.

[9] Hidalgo CG, Chung CS, Saripalli C (2013). The multifunctional Ca^2+^/calmodulin-dependent protein kinase II delta (CaMKIIδ) phosphorylates cardiac titin’s spring elements. *J. Mol. Cell Cardiol.*.

[10] Levy De, Wang DX, Lu Q (2008). Aryl–indolyl maleimides as inhibitors of CaMKIIδ. Part 1: SAR of the aryl region. *Bioorg. Med. Chem. Lett.*.

[11] Mavunkel B, Xu YJ, Goyal B (2008). Pyrimidine-based inhibitors of CaMKIIδ. *Bioorg. Med. Chem. Lett.*.

[12] Liu Q, Molkentin JD (2011). Protein kinase Cα as a heart failure therapeutic target. *J. Mol. Cell. Cardiol.*.

[13] Walker JW (2006). Protein kinase C, troponin I and heart failure: overexpressed, hyperphosphorylated and underappreciated?. *J. Mol. Cell. Cardiol.*.

[14] Niizuma S, Inuzuka Y, Okuda J (2012). Effect of persistent activation of phosphoinositide 3-kinase on heart. *Life Sci.*.

[15] Rose BA, Force T, Wang Y (2010). Mitogen-activated protein kinase signaling in the heart: angels versus demons in a heart-breaking tale. *Physiol. Rev.*.

[16] Abraham ST, Benscoter HA, Schworer CM, Schworer CM, Singer HA (1997). A role for Ca^2+^/calmodulin-dependent protein kinase II in the mitogen-activated protein kinase signaling cascade of cultured rat aortic vascular smooth muscle cells. *Circ. Res.*.

[17] Hidalgo CG, Chung CS, Saripalli C (2013). The multifunctional Ca(^2+^)/calmodulin-dependent protein kinase II delta (CaMKIIdelta) phosphorylates cardiac titin’s spring elements. *J. Mol. Cell. Cardiol.*.

[18] Picht E, Desantiago J, Huke S, Kaetzel MA, Dedman JR, Bers DM (2007). CaMKII inhibition targeted to the sarcoplasmic reticulum inhibits frequency dependent acceleration of relaxation and Ca(^2+^) current facilitation. *J. Mol. Cell. Cardiol.*.

[19] Swulius MT, Waxham MN (2008). Ca(2+)/calmodulin-dependent protein kinases. *Cell. Mol. Life Sci.*.

[20] Barabutis N, Verin A, Catravas JD (2016). Regulation of pulmonary endothelial barrier function by kinases. *Am. J. Physiol. Lung Cell. Mol. Physiol.*.

[21] Mayadevi M, Sherin DR, Keerthi VS, Rajasekharan KN, Omkumar RV (2012). Curcumin is an inhibitor of calcium/calmodulin dependent protein kinase II. *Bioorg. Med. Chem.*.

[22] Mayadevi M, Sherin DR, Keerthi VS, Rajasekharan KN, Omkumar RV (2012). Curcumin is an inhibitor of calcium/calmodulin dependent protein kinase II. *Bioorg. Med. Chem.*.

[23] Tokumitsu H, Chijiwa T, Hagiwara M, Mizutani A, Terasawa M, Hidaka H (1990). KN-62, 1-[*N*,O-bis(5-isoquinolinesulfonyl)-*N*-methyl-L-tyrosyl]-4-phenylpiperazine, a specific inhibitor of Ca^2+^/calmodulin-dependent protein kinase II. *J. Biol. Chem.*.

[24] Davies SP, Reddy H, Caivano M, Cohen P (2000). Specificity and mechanism of action of some commonly used protein kinase inhibitors. *Biochem. J.*.

[25] Yokokura H, Okada Y, Terada O, Hidaka H (1996). HMN-709, a chlorobenzenesulfonamide derivative, is a new membrane-permeable calmodulin antagonist. *Jpn J. Pharmacol.*.

[26] Lu Q, Chen Z, Perumattam J (2008). Aryl–indolyl maleimides as inhibitors of CaMKIIδ. Part 3: importance of the indole orientation. *Bioorg. Med. Chem. Lett.*.

[27] Komiya M, Asano S, Koike N (2012). Synthesis and structure based optimization of 2-(4-phenoxybenzoyl)-5-hydroxyindole as a novel CaMKII inhibitor. *Bioorg. Med. Chem.*.

[28] Beauverger P, Gegis G, Biscarrat S, Duclos O, Mccort G. (2012). 5-Oxo-5,8-dihydropyridol [2,3-d] pyrimidine derivatives as CAMKII kinase inhibitors for treating cardiovascular disease.

[29] Shahin R, Taha MO (2012). Elaborate ligand-based modeling and subsequent synthetic exploration unveil new nanomolar Ca^2+^/calmodulin-dependent protein kinase II inhibitory leads. *Bioorg. Med. Chem.*.

[30] Shahin R, Taha MO (2012). Elaborate ligand-based modeling and subsequent synthetic exploration unveil new nanomolar Ca^2+^/calmodulin-dependent protein kinase II inhibitory leads.

[31] Erickson JR (2014). Mechanisms of CaMKII activation in the heart. *Front. Pharmacol.*.

[32] Knight ZA, Shokat KM (2005). Features of selective kinase inhibitors. *Chem. Biol.*.

[33] Wu P, Clausen MH, Nielsen TE (2015). Allosteric small-molecule kinase inhibitors. *Pharmacol. Ther.*.

[34] Oh KS, Oh BK, Park CH (2013). Cardiovascular effects of a novel selective Rho kinase inhibitor, 2-(1H-indazole-5-yl)amino-4-methoxy-6-piperazino triazine (DW1865). *Eur. J. Pharmacol.*.

[35] Noma K, Oyama N, Liao JK (2006). Physiological role of ROCKs in the cardiovascular system. *Am. J. Phsyiol. Cell Physiol.*.

[36] Schroter T, Minond D, Weiser A (2008). Comparison of miniaturized time-resolved fluorescence resonance energy transfer and enzyme-coupled luciferase high-throughput screening assays to discover inhibitors of Rho-kinase II (ROCK-II). *J. Biomol. Screen.*.

[37] Feng Y, Yin Y, Weiser A (2008). Discovery of substituted 4-(pyrazol-4-yl)-phenylbenzodioxane-2-carboxamides as potent and highly selective Rho kinase (ROCK-II) inhibitors. *J. Med. Chem.*.

[38] Ono-Saito N, Niki I, Hidaka H (1999). H-series protein kinase inhibitors and potential clinical applications. *Pharmacol. Ther.*.

[39] Uehata M, Ishizaki T, Satoh H (1997). Calcium sensitization of smooth muscle mediated by a rho-associated protein kinase in hypertension. *Nature*.

[40] Feng Y, Lograsso PV, Defert O, Li R (2016). Rho kinase (ROCK) inhibitors and their therapeutic potential. *J. Med. Chem.*.

[41] Seiler RW, Steiger HJ (2001). *Cerebral Vasospasm.*.

[42] Mueller BK, Mack H, Teusch N (2005). Rho kinase, a promising drug target for neurological disorders. *Nat. Rev. Drug Discov.*.

[43] Garnock-Jones KP (2014). Ripasudil: first global approval. *Drugs*.

[44] Tanihara H, Inoue T, Yamamoto T (2013). Phase II randomized clinical study of a rho kinase inhibitor, K-115, in primary open-angle glaucoma and ocular hypertension. *Am. J. Ophthalmol.*.

[45] Tanihara H, Inoue T, Yamamoto T (2013). Phase II clinical trials of a selective rho kinase inhibitor, K-115. *JAMA Ophthalmol.*.

[46] Ikenoya M, Hidaka H, Hosoya T, Suzuki M, Yamamoto N, Sasaki Y (2002). Inhibition of rho-kinase-induced myristoylated alanine-rich C kinase substrate (MARCKS) phosphorylation in human neuronal cells by H-1152, a novel and specific rho-kinase inhibitor. *J. Neurochem.*.

[47] Sasaki Y, Suzuki M, Hidaka H (2002). The novel and specific rho-kinase inhibitor (S)-(+)-2-methyl-1-[(4-methyl-5-isoquinoline)sulfonyl]-homopiperazine as a probing molecule for rho-kinase-involved pathway. *Pharmacol. Ther.*.

[48] Lavogina D, Kalind K, Bredihhina J (2012). Conjugates of 5-isoquinolinesulfonylamides and oligo-D-arginine possess high affinity and selectivity towards Rho kinase (ROCK). *Bioorg. Med. Chem. Lett.*.

[49] Ishizaki T, Uehata M, Tamechika I (2000). Pharmacological properties of Y-27632, a specific inhibitor of rho-associated kinases. *Mol. Pharmacol.*.

[50] Tokushige H, Waki M, Takayama Y, Tanihara H (2011). Effects of Y-39983, a selective rho-associated protein kinase inhibitor, on blood flow in optic nerve head in rabbits and axonal regeneration of retinal ganglion cells in rats. *Curr. Eye Res.*.

[51] Feng Y, Cameron MD, Frackowiak B (2007). Structure–activity relationships, and drug metabolism and pharmacokinetic properties for indazole piperazine and indazole piperidine inhibitors of ROCK-II. *Bioorg. Med. Chem. Lett.*.

[52] Feng Y, Yin Y, Weiser A (2008). Discovery of substituted 4-(pyrazol-4-yl)-phenylbenzodioxane-2-carboxamides as potent and highly selective rho kinase (ROCK-II) inhibitors. *J. Med. Chem.*.

[53] Fang X, Yin Y, Wang B (2010). Tetrahydroisoquinoline derivatives as potent and selective rho kinase inhibitors. *J. Med. Chem.*.

[54] Mimi L, Quan Z, Wang C (2015). Tricyclic pyrido-carboxamide derivatives as rock inhibitors WO 2015002926 A1.

[55] Mishra RK, Alokam R, Singhal SM (2014). Design of novel rho kinase inhibitors using energy based pharmacophore modeling, shape-based screening, *in silico* virtual screening, and biological evaluation. *J. Chem. Inf. Model.*.

[56] Oh KS, Oh BK, Park CH (2013). Cardiovascular effects of a novel selective rho kinase inhibitor, 2-(1H-indazole-5-yl)amino-4-methoxy-6-piperazino triazine (DW1865). *Eur. J. Pharmacol.*.

[57] Shahin R, Alqtaishat S, Taha MO (2012). Elaborate ligand-based modeling reveal new submicromolar Rho kinase inhibitors. *J. Comput. Aided Mol. Des.*.

[58] Feng Y, Lograsso P (2014). Rho kinase inhibitors: a patent review (2012–2013). *Expert Opin. Ther. Pat.*.

[59] Feng Y, Lograsso PV, Defert O, Li R (2016). Rho kinase (ROCK) inhibitors and their therapeutic potential. *J. Med. Chem.*.

[60] Churchill E, Budas G, Vallentin A, Koyanagi T, Mochly-Rosen D (2008). PKC isozymes in chronic cardiac disease: possible therapeutic targets?. *Annu. Rev. Pharmacol. Toxicol.*.

[61] Koide Y (2003). Differential induction of protein kinase C isoforms at the cardiac hypertrophy stage and congestive heart failure stage in Dahl salt-sensitive rats. *Hypertens. Res.*.

[62] Liu Q, Molkentin JD (2011). Protein kinase Cα as a heart failure therapeutic target. *J. Mol. Cell. Cardiol.*.

[63] Mochly-Rosen D (2000). Cardiotrophic effects of protein kinase C epsilon: analysis by *in vivo* modulation of PKC[epsiv] translocation. *Circ. Res.*.

[64] Ferreira JC (2011). Pharmacological inhibition of βIIPKC is cardioprotective in late-stage hypertrophy. *J. Mol. Cell. Cardiol.*.

[65] Ferreira JC, Boer BN, Grinberg M, Brum PC, Mochly-Rosen D (2012). Protein quality control disruption by PKCβII in heart failure; rescue by the selective PKCβII inhibitor, βIIV5–3. *PLoS ONE*.

[66] Ferreira JC, Brum PC, Mochly-Rosen D (2011). βIIPKC and ϵPKC isozymes as potential pharmacological targets in cardiac hypertrophy and heart failure. *J. Mol. Cell. Cardiol.*.

[67] Omura S, Iwai Y, Hirano A (1977). A new alkaloid AM-2282 of *Streptomyces* origin. Taxonomy, fermentation, isolation and preliminary characterization. *J. Antiobiot. (Tokyo)*.

[68] Radhika P, Kumar MMK, Nagasree KP, Rahman AUr (2015). Protein kinase inhibitors from microorganisms. *Studies in Natural Products Chemistry.*.

[69] Koshino H, Osada H, Isono K (1992). A new inhibitor of protein kinase C, RK-1409 (7-oxostaurosporine). II. Fermentation, isolation, physico-chemical properties and structure. *J. Antiobiot. (Tokyo)*.

[70] Osada H, Koshino H, Kudo T, Onose R, Isono K (1992). A new inhibitor of protein kinase C, RK-1409 (7-oxostaurosporine). I. Taxonomy and biological activity. *J. Antiobiot. (Tokyo)*.

[71] Kobayashi E, Nakano H, Morimoto M, Tamaoki T (1989). Calphostin C (UCN-1028C), a novel microbial compound, is a highly potent and specific inhibitor of protein kinase C. *Biochem. Biophys. Res. Commun.*.

[72] Wagner J, Von Matt P, Sedrani R (2009). Discovery of 3-(1H-indol-3-yl)-4-[2-(4-methylpiperazin-1-yl)quinazolin-4-yl]pyrrole-2,5-dione (AEB071), a potent and selective inhibitor of protein kinase C isotypes. *J. Med. Chem.*.

[73] Van Eis MJ, Evenou JP, Floersheim P (2011). 2,6-Naphthyridines as potent and selective inhibitors of the novel protein kinase C isozymes. *Bioorg. Med. Chem. Lett.*.

[74] Li H, Hong Y, Nukui S (2011). Identification of novel pyrrolopyrazoles as protein kinase C beta II inhibitors. *Bioorg. Med. Chem. Lett.*.

[75] Mochly-Rosen D, Das K, Grimes KV (2012). Protein kinase C, an elusive therapeutic target?. *Nat. Rev. Drug Discov.*.

[76] Packer M (1993). Double-blind, placebo-controlled study of the efficacy of flosequinan in patients with chronic heart failure. Principal Investigators of the REFLECT Study. *J. Am. Coll. Cardiol.*.

[77] (2007). Ruboxistaurin: LY 333531. *Drugs R D*.

[78] Poli A, Mongiorgi S, Cocco L, Follo MY (2014). Protein kinase C involvement in cell cycle modulation. *Biochem. Soc. Trans.*.

[79] Li H (2006). Protein kinase C: novel isozyme-selective peptide inhibitors. *Exp. Opin. Therap. Pat.*.

[80] Alam MA, Uddin SJ, Brown L, Rahman AUr (2012). Mitogen-activated protein kinase and natural phenolic compounds in cardiovascular remodeling. *Studies in Natural Products Chemistry.*.

[81] Goetze S, Xi XP, Kawano Y (1999). TNF-alpha-induced migration of vascular smooth muscle cells is MAPK dependent. *Hypertension*.

[82] Willette RN, Eybye ME, Olzinski AR (2009). Differential effects of p38 mitogen-activated protein kinase and cyclooxygenase 2 inhibitors in a model of cardiovascular disease. *J. Pharmacol. Exp. Ther.*.

[83] Ren J, Zhang S, Kovacs A, Wang Y, Muslin AJ (2005). Role of p38 alpha MAPK in cardiac apoptosis and remodeling after myocardial infarction. *J. Mol. Cell. Cardiol.*.

[84] Chen C, Yu R, Owuor ED, Kong AN (2000). Activation of antioxidant-response element (ARE), mitogen-activated protein kinases (MAPKs) and caspases by major green tea polyphenol components during cell survival and death. *Arch. Pharm. Res.*.

[85] Cuenda A, Rouse J, Doza YN (1995). SB 203580 is a specific inhibitor of a MAP kinase homologue which is stimulated by cellular stresses and interleukin-1. *FEBS Lett.*.

[86] Jackson JR, Bolognese B, Hillegass L (1998). Pharmacological effects of SB 220025, a selective inhibitor of P38 mitogen-activated protein kinase, in angiogenesis and chronic inflammatory disease models. *J. Pharmacol. Exp. Ther.*.

[87] Wadsworth SA, Cavender DE, Beers SA (1998). RWJ 67657, a potent, orally active inhibitor of p38 mitogen-activated protein kinase. *J. Pharmacol. Exp. Ther.*.

[88] Clinical Trials Database (2015). NCT00996840.

[89] Laufer SA, Wagner GK, Kotschenreuther DA, Albrecht W (2003). Novel substituted pyridinyl imidazoles as potent anticytokine agents with low activity against hepatic cytochrome P450 enzymes. *J. Med. Chem.*.

[90] Das J, Moquin RV, Pitt S (2008). Pyrazolo-pyrimidines: A novel heterocyclic scaffold for potent and selective p38α inhibitors. *Bioorg. Med. Chem. Lett*.

[91] Clinical Trials Database (2015). NCT00570752.

[92] Clinical Trials Database (2015). NCT00399906.

[93] Collis AJ, Foster ML, Halley F (2001). RPR203494 a pyrimidine analogue of the p38 inhibitor RPR200765A with an improved *in vitro* potency. *Bioorg. Med. Chem. Lett.*.

[94] Haddad JJ (2001). VX-745. Vertex Pharmaceuticals. *Curr. Opin. Investig. Drugs*.

[95] Sokol L, Cripe L, Kantarjian H (2013). Randomized, dose-escalation study of the p38 alpha MAPK inhibitor SCIO-469 in patients with myelodysplastic syndrome. *Leukemia*.

[96] Skurk C, Maatz H, Rocnik E, Bialik A, Force T, Walsh K (2005). Glycogen-synthase kinase3beta/beta-catenin axis promotes angiogenesis through activation of vascular endothelial growth factor signaling in endothelial cells. *Circ. Res.*.

[97] Juhaszova M, Zorov DB, Yaniv Y, Nuss HB, Wang S, Sollott SJ (2009). Role of glycogen synthase kinase-3beta in cardioprotection. *Circ. Res.*.

[98] Meijer L, Thunnissen AM, White AW (2000). Inhibition of cyclin-dependent kinases, GSK-3beta and CK1 by hymenialdisine, a marine sponge constituent. *Chem. Biol.*.

[99] Leclerc S, Garnier M, Hoessel R (2001). Indirubins inhibit glycogen synthase kinase-3 beta and CDK5/p25, two protein kinases involved in abnormal tau phosphorylation in Alzheimer’s disease. A property common to most cyclin-dependent kinase inhibitors?. *J. Biol. Chem.*.

[100] Leost M, Schultz C, Link A (2000). Paullones are potent inhibitors of glycogen synthase kinase-3beta and cyclin-dependent kinase 5/p25. *Eur. J. Biochem.*.

[101] Mettey Y, Gompel M, Thomas V (2003). Aloisines, a new family of CDK/GSK-3 inhibitors. SAR study, crystal structure in complex with CDK2, enzyme selectivity, and cellular effects. *J. Med. Chem.*.

[102] Zhang HC, White KB, Ye H (2003). Macrocyclic bisindolylmaleimides as inhibitors of protein kinase C and glycogen synthase kinase-3. *Bioorg Med. Chem. Lett.*.

[103] Duan Q, Madan ND, Wu J (2015). Role of phosphoinositide 3-kinase IA (PI3K-IA) activation in cardioprotection induced by ouabain preconditioning. *J. Mol. Cell. Cardiol.*.

[104] Pretorius L, Du XJ, Woodcock EA (2001). Reduced phosphoinositide 3-kinase (p110 alpha) activation increases the susceptibility to atrial fibrillation. *Am. J. Pathol.*.

[105] Mcmullen JR, Amirahmadi F, Woodcock EA (2007). Protective effects of exercise and phosphoinositide 3-kinase(p110 alpha) signaling in dilated and hypertrophic cardiomyopathy. *Proc. Natl Acad. Sci. USA*.

[106] Jasiński M, Jasińska L, Ogrodowczyk M (2013). Resveratrol in prostate diseases – a short review. *Central European J. Urol.*.

[107] Chong E, Chang SL, Hsiao YW (2015). Resveratrol, a red wine antioxidant, reduces atrial fibrillation susceptibility in the failing heart by PI3K/AKT/eNOS signaling pathway activation.. *Heart Rhythm.*.

[108] Liu P, Cheng H, Roberts TM, Zhao JJ (2009). Targeting the phosphoinositide 3-kinase (PI3K) pathway in cancer. *Nat. Rev. Drug Discov.*.

[109] Zhang J, Fan G, Zhao H (2017). Targeted inhibition of focal adhesion kinase attenuates cardiac fibrosis and preserves heart function in adverse cardiac remodeling. *Sci. Rep.*.

[110] Fang Y, Wang D, Xu X (2017). Synthesis, biological evaluation, and molecular dynamics (MD) simulation studies of three novel F-18 labeled and focal adhesion kinase (FAK) targeted 5-bromo pyrimidines as radiotracers for tumor. *Eur. J. Med. Chem.*.

